# Coronary microvascular dysfunction in autoimmune rheumatic diseases: beyond coronary flow velocity reserve

**DOI:** 10.3389/fcvm.2024.1372703

**Published:** 2024-08-21

**Authors:** Annagrazia Cecere, Martina Perazzolo Marra, Elisabetta Zanatta, Giovanni Civieri, Sabino Iliceto, Francesco Tona

**Affiliations:** ^1^ Department of Cardiac, Thoracic, and Vascular Sciences and Public Health, University of Padova, Padova, Italy; ^2^ Department of Medicine, University of Padova, Padova, Italy

**Keywords:** coronary microvascular dysfunction, autoimmune rheumatic diseases, coronary flow reserve, cardiac magnetic resonance, coronary flow velocity reserve

## Abstract

Autoimmune rheumatic diseases (ARDs) are a heterogeneous group of disorders characterized by an inappropriate immune reactivity against different body tissues. Patients affected by ARDs present increased cardiovascular morbidity and mortality, which significantly impacts long-term prognosis. Endothelial dysfunction, inflammation, oxidative stress, and autoimmunity are strictly involved in atherosclerosis progression and coronary microvascular dysfunction (CMD), both of which contribute to increased cardiovascular risk. CMD represents the inability of the coronary microvasculature to respond with vasodilation to increased cardiac metabolic demands and can be assessed by non-invasive and invasive imaging tests. Coronary flow velocity reserve assessed by echocardiography has been demonstrated to accurately identify ARDs patients with CMD. However, stress cardiac magnetic resonance (CMR) accurately assesses myocardial ischemia, perfusion, and viability in ARDs patients. The myocardial perfusion reserve index (MPRI) is a robust semiquantitative imaging marker that represents the vasodilatory capacity of the coronary microcirculation in response to a vasodilator stress. In the absence of significant coronary stenosis, ARDs patients revealed a reduced MPRI in comparison with the general population, regardless of the presence of myocardial fibrosis. Identification of CMD in asymptomatic patients could be crucial to precociously start targeted medical therapy, avoiding major adverse cardiac events in this clinical setting. This review aims to summarize the current evidence regarding CMD in ARDs patients, focusing on the role of stress CMR and the promising myocardial perfusion analysis.

## Autoimmune rheumatic diseases

Autoimmune rheumatic diseases (ARDs) include a heterogeneous group of disorders characterized by an impairment of tolerance to self-antigens and/or immunoregulation, responsible for an inappropriate immune reactivity against different body tissues (1). In particular, ARDs include rheumatoid arthritis (RA), spondyloarthropathies, systemic lupus erythematosus (SLE), systemic vasculitis, inflammatory cardiomyopathies, mixed connective tissue diseases, and systemic sclerosis (SS). In recent years, the introduction of novel targeted therapy for ARDs has reduced disease-related mortality, although in the absence of a relevant impact on long-term prognosis. In fact, although the average 5-year survival rate in ARDs patients under optimized treatment is currently similar to the general population (2), the long-term life expectancy is significantly lower (3). Interestingly, the reduced long-term life expectancy cannot be attributed to the progression of rheumatic illness but to cardiovascular diseases (CVD), which significantly impact the patient's prognosis (4). In ARDs patients, CVD could be responsible for not only accelerated atherosclerosis but also coronary microvascular dysfunction (CMD) (5). Moreover, CVD in ARDs patients could be asymptomatic or with few symptoms for a long time and become clinically overt after many years with a poor prognosis (6).

## Coronary microvascular dysfunction

Coronary microcirculation guarantees the correct blood flow, according to the oxygen requirement, by regulating the resistance of the vascular component (7). The large epicardial coronary arteries (500 µm–5 mm in diameter) are conductance vessels, offering very little resistance. Their main role, secondary to the endothelium-dependent dilatation, is to transport adequate blood quantity. Conversely, pre-arterioles and arterioles control the coronary blood flow, representing the coronary microvasculature. In particular, the epicardial pre-arterioles (100–500 µm in diameter) play as a “pressure controller” at the origin of the arterioles and respond to flow-related stimuli with endothelium-dependent vasoreactivity. The intramyocardial arterioles (<100 µm in diameter) have the highest resistance and, depending on the vessel size, respond by myogenic control or metabolites. In fact, medium-sized arterioles (40–100 µm in diameter) present stretch receptors in the vascular smooth muscle cells and react to pressure variations, leading to vasoconstriction when the intraluminal pressure increases and, conversely, to vasodilation when the pressure decreases. On the contrary, the small arterioles (<40 µm in diameter) are responsive to the intramyocardial concentration of metabolites. Therefore, an increased metabolic activity leads to vasodilation, responsible for pressure reduction in medium-sized arterioles, myogenic dilation, and subsequently increased flow upstream. Finally, in response to the endothelium-dependent vasodilation, pre-arteriole and epicardial coronary artery dilation occurs (8). Capillaries and venules represent the final part of the coronary circulation, and, as well as epicardial arteries, they act as capacitance vessels. This final part of the coronary circulation is crucial for the exchange of oxygen, nutrients, and metabolites between blood and myocardial tissue. Morpho-functional abnormalities of the coronary microcirculation could lead to inadequate blood and oxygen transport, contributing to the pathogenesis of myocardial ischemia (9).

CMD is due to the incompetence of the coronary microvasculature to respond to the increased cardiac metabolic requests (10). Thus, it could be due to the inability to increase coronary blood flow because of functional impairment, the structural damage of the coronary microcirculation (vasodilatory abnormality), and/or the reduction of coronary blood flow (coronary microvascular vasospasm) (8). Thus, this condition could be secondary to cardiac or systemic conditions, responsible for left ventricular (LV) hypertrophy (hypertrophic cardiomyopathy, aortic stenosis) (11), or to diseases related to chronic inflammation (10, 12, 13).

Clinically, patients with CMD present exercise-related angina, evidence of ischemia in non-invasive tests, and either no stenosis or no functionally relevant coronary stenosis (14, 15). Myocardial contractility evaluation could help clinicians in the differential diagnosis between coronary artery disease (CAD) and CMD. An epicardial coronary stenosis, in fact, typically produces a localized myocardial perfusion impairment with a segmental reduction of LV contractility. Conversely, in patients with CMD, the myocardial perfusion impairment is usually global without segmental wall motion abnormalities, because it is not related to a single coronary artery (16). Consequently, CMD patients present a preserved or slightly reduced LV systolic function. Although the absence of obstructive epicardial coronary stenosis, CMD, more frequent in female patients, represents an important cause of myocardial ischemia and is associated with a greater risk for major adverse cardiovascular events (MACEs) (17–20). Nevertheless, due to the similarity with angina symptoms, microvascular angina could be diagnosed only after the exclusion of an obstructive epicardial coronary stenosis.

## Mechanisms contributing to CMD in ARDs

The long-term prognosis of ARDs patients is closely related to CVD. As reported by Shinomiya et al. (21) in a Japanese population, the prognostic impact of CVD changed dramatically during the last years, making malignancy the most common cause of mortality in RA patients. Similarly, the increased CV risk documented in patients with ankylosing spondylitis, psoriatic arthritis, and SLE (22–24) has not been completely attributed to CAD. Thus, CMD has been advocated as a possible explanation of the increased CV risk in ARDs patients, sharing some pathophysiological determinants with ischemic disease.

The pathogenesis of CMD in ARDs has not been fully addressed and remains a debated topic of investigation (8). The clinical inflammation burden in arthritis patients demonstrated to be associated with microvascular flow impairment (25). Accordingly, the use of anti-inflammatory biological therapies, such as antitumor necrosis factor-α (TNF-α) treatments, has been shown to improve coronary and peripheral microvascular dysfunction (26). On the other side, although influenced by the observational nature of the study and the inflammation burden evaluation, the longitudinal Dudley Rheumatoid Arthritis Comorbidity Cohort (DRACCO) study did not evidence any correlation between cumulative inflammatory burden and endothelial function in a 6-year follow-up (27). In addition, a recent meta-analysis (28) across over 20 studies revealed that coronary flow reserve (CFR) in ARDs, although lower than that in the general population, seems not to be related to inflammation, dyslipidemia, obesity, age, or arterial blood pressure. Therefore, the role of inflammation in determining CMD in ARDs is still controversial.

Endothelial dysfunction represents the *primum movens* in the microcirculatory impairment, as well as in the atherosclerotic process, and it is due to an imbalance between vasodilation and vasoconstrictive release factors (8, 29). Endothelial dysfunction and arterial stiffness have been described in many chronic inflammatory conditions, including inflammatory bowel disease and psoriasis (30, 31). Endothelial dysfunction is common in ARDs patients, playing a crucial role in both macro- and microvascular dysfunction (10, 32, 33). Systemic endothelial dysfunction is closely related to both reduced availability of nitric oxide (NO) and increased production of reactive oxygen species, secondary to oxidative stress (34). In fact, increased levels of inflammatory mediators, such as interleukin-17 (IL-17), interferon-γ (INF-γ), and TNF-α, activated NADPH oxidases (Nox) enzymes and increased reactive oxygen species production, acting a pivotal role in the pathogenesis of arthritis and endothelial dysfunction (34). Haruna et al. (35) demonstrated that angiotensin receptor blockers inhibit Nox expression, improving endothelial function in animal models of arthritis. Therefore, oxidative stress could be responsible for both local and systemic RA-related vascular damage.

In addition to the pathogenesis of ARDs, T-cells, natural killer (NK) cells, and monocytes play a role also in endothelial dysfunction and CMD in rheumatic diseases (36). NK cells promote vasoconstriction of arterioles and could dysregulate CD28 null (CD4+ and CD8+), producing pro-inflammatory cytokines (TNF-α, INF-γ, IL-2) involved in oxidative stress, endothelial dysfunction, and arteriolar rarefaction (37). Moreover, lymphocyte activation could determine oxidative stress, playing a key role in the pathogenesis and vascular dysfunction of ARDs patients. T-cells and antigen-presenting cells express Nox2 that mediates their activation and immune functions (38). Finally, overexpression of pro-inflammatory cytokines (IL-18, IL-33, and TNF-α) has been identified in RA patients with vascular impairment, confirming the key role in both the inflammatory process and the development of endothelial dysfunction (39). Finally, an imbalance in sympathetic/parasympathetic activation can determine motility dysfunction, acting directly on vascular smooth cells (40).

## Assessment of coronary microvascular dysfunction

**Figure 1:**
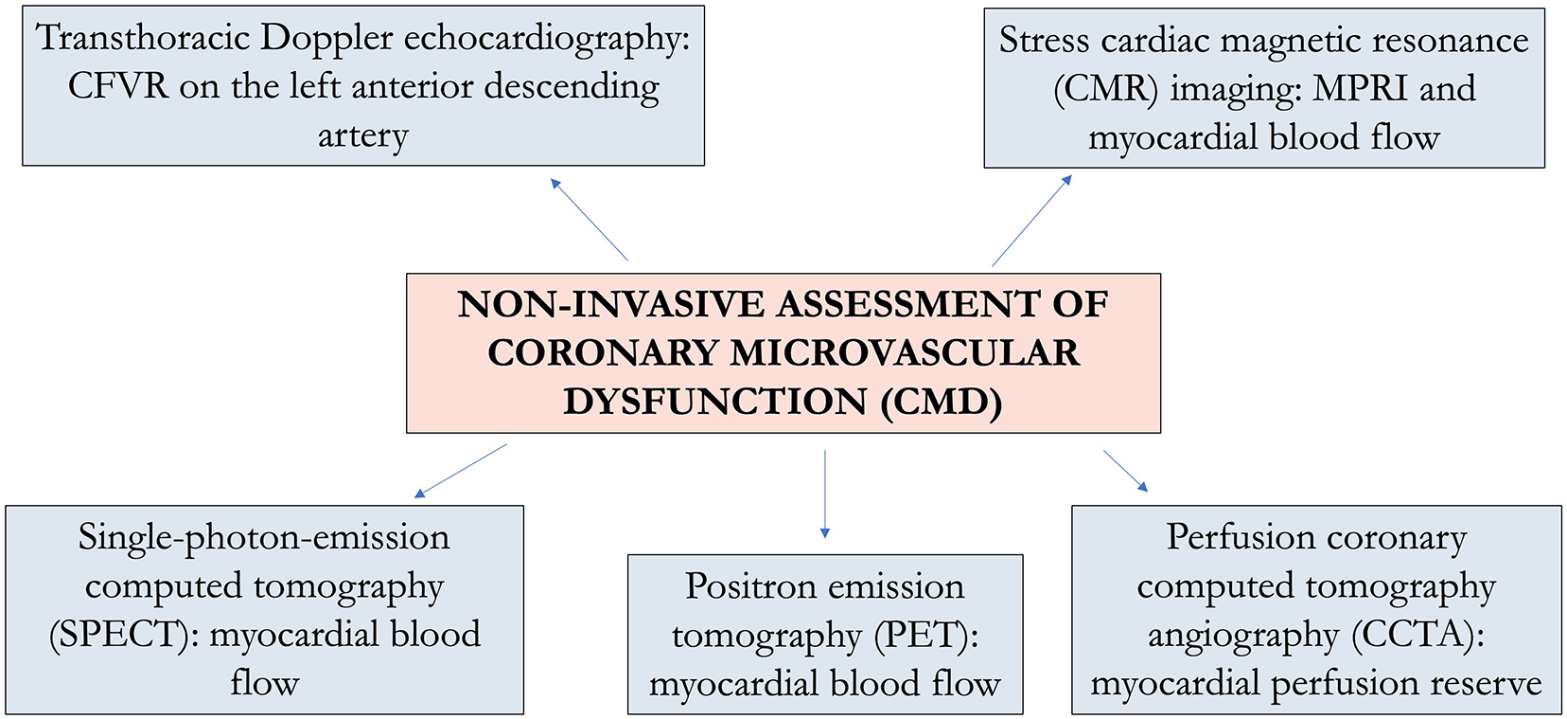
Non-invasive assessment of coronary microvascular dysfunction.

Based on the functional assessment of the coronary arteries, CMD diagnosis could be performed with invasive and non-invasive methods [(14, 15), Figure 1]. Echocardiography demonstrated to properly evaluate the endothelium-independent microvascular function with the assessment of coronary flow velocity reserve (CFVR) on the left anterior descending artery (41). CFR, first introduced by Gould in 1974, describes the ability of coronary flow to respond with dilation to an increase in metabolic requirements (42, 43). As a dimensionless value, CFVR is defined by the ratio between hyperemic and basal diastolic coronary blood flow velocity (44). During stress (physical or pharmacological-induced with vasodilators), this ratio may increase up to five times the resting values (43). This crucial parameter has been demonstrated to be strongly related to coronary artery lesion severity angiographically detected and intracoronary Doppler flow wire measurements in ischemic heart disease (41). A value of CFVR ≤2.5 is considered abnormal, and it could be due to an epicardial coronary stenosis or myocardial bridge and to CMD (45, 46). In particular, in the absence of an epicardial coronary artery stenosis or bridge, a reduced CFVR is an expression of CMD, and it could be related to (1) a reduced peripheral resistance in basal condition, responsible for an increased coronary flow at baseline and/or (2) high hyperemic peripheral resistance that reducing the arteriolar vasodilatory capability (7). In ARDs patients, a CFVR of ≤2.5 has been demonstrated to correctly identify CMD in the absence of epicardial coronary stenosis (47, 48).

Cardiac magnetic resonance (CMR) represents a non-invasive method to detect chronic perfusion defects, inducible ischemia, and CMD with vasodilator administration (49).

Single-photon emission computed tomography (SPECT) evaluates the differences in the radionuclide distribution, in the different regions of myocardium, before and after stress (50). Thus, SPECT could detect microvascular impairment through the identification of the relative blood perfusion to the different regions of the myocardium. Moreover, due to the limitations of radiotracers, characterized by low first-pass extraction, significant roll-off uptake at higher flow rates and intestinal uptake, and poor camera sensitivity and temporal resolution, the use of SPECT is quite limited (51). Conversely, positron emission tomography (PET) with 18 F-fluorodeoxyglucose can determine the absolute myocardial blood flow (ml/gr/min), allowing an accurate and precise CMD assessment (50). The myocardial perfusion reserve assessed by PET has been demonstrated to be accurate and reproducible (52, 53). In addition, this parameter has been correlated to adverse outcomes, suggesting a possible prognostic role in the risk stratification of ischemic patients (54). Due to the possibility of detecting and monitoring myocardial inflammation, the combined use of PET-CMR resulted particularly useful in inflammatory cardiomyopathy, such as sarcoidosis (55). However, similarly to SPECT, PET has some limitations as well regarding radiation exposure, radiotracer cost, and diffusion of the exam (56).

Finally, perfusion coronary computed tomography angiography (CCTA) revealed its non-inferiority to SPECT in myocardial inducible ischemia detection (57). Similar to CMR, perfusion CCTA, using pharmacological stressors, accurately identifies the presence of a hypodense area due to reduced perfusion during hyperemia (58). However, due to the heterogeneity of evidence in the literature regarding pharmacologic stress agents, imaging sequence acquisition and post-processing, and different scanner machines used, an expert consensus regarding the use and feasibility of perfusion CCTA is lacking. Therefore, based on the high negative predictive value of CCTA, the addition of perfusion evaluation to the standard CCTA protocol acquisition could be considered in those patients with coronary stenosis with unknown hemodynamic significance (59, 60).

Accordingly, the European Guidelines on chronic coronary syndrome suggested with an indication IIb the non-invasive assessment of CFR with transthoracic echocardiography on the left anterior descending artery, CMR, and PET (14).

Finally, CMD could be also assessed invasively with coronary angiography. The first invasive evidence of CMD was the observation of “slow” coronary flow in patients with chest pain and no obstructive coronary lesions (61). In the absence of a significant coronary lesion, the slow flow was attributed to the high coronary microvasculature resistance, which delayed the contrast passage in the distal part of the coronary. It is possible to quantify the contrast agent velocity passage in the coronary artery. In fact, the thrombolysis in myocardial infarction (TIMI) criteria evaluates the grade of opacification after contrast administration, assigning a score from 0, no reperfusion, to 3, optimal reperfusion. Secondly, the corrected TIMI frame count counts the number of cine frame numbers needed by the contrast agent to reach standardized distal coronary landmarks (62). In addition, CMD could be assessed invasively through the index of myocardial resistance, based on the hyperemic and basal intracoronary pressure (63), reflecting functional and/or structural coronary abnormalities (8). Finally, the intracoronary administration of vasoactive agents (acetylcholine or ergonovine) could assess the endothelium-dependent microvascular function. These vasoactive agents produce a massive stimulation of NO, resulting in vasodilation in normal coronary arteries. On the contrary, in CMD patients, the administration of vasoactive agents cannot contrast the vasoconstriction, induced by the endothelial dysfunction (64).

## Stress CMR in the CMD evaluation: from acquisition to interpretation

Stress CMR has been demonstrated to accurately assess myocardial ischemia and viability, as well as CMD, revealing a good correlation with PET (65). In comparison to other non-invasive imaging tests, stress CMR presents some technical advantages, principally connected to its high spatial resolution and excellent safety profile, without the use of ionizing radiation or iodinated contrast agents (65–67). Finally, the complete independence from the patient's acoustic window and soft tissue attenuation makes CMR very promising in the CMD evaluation.

**Figure 2:**
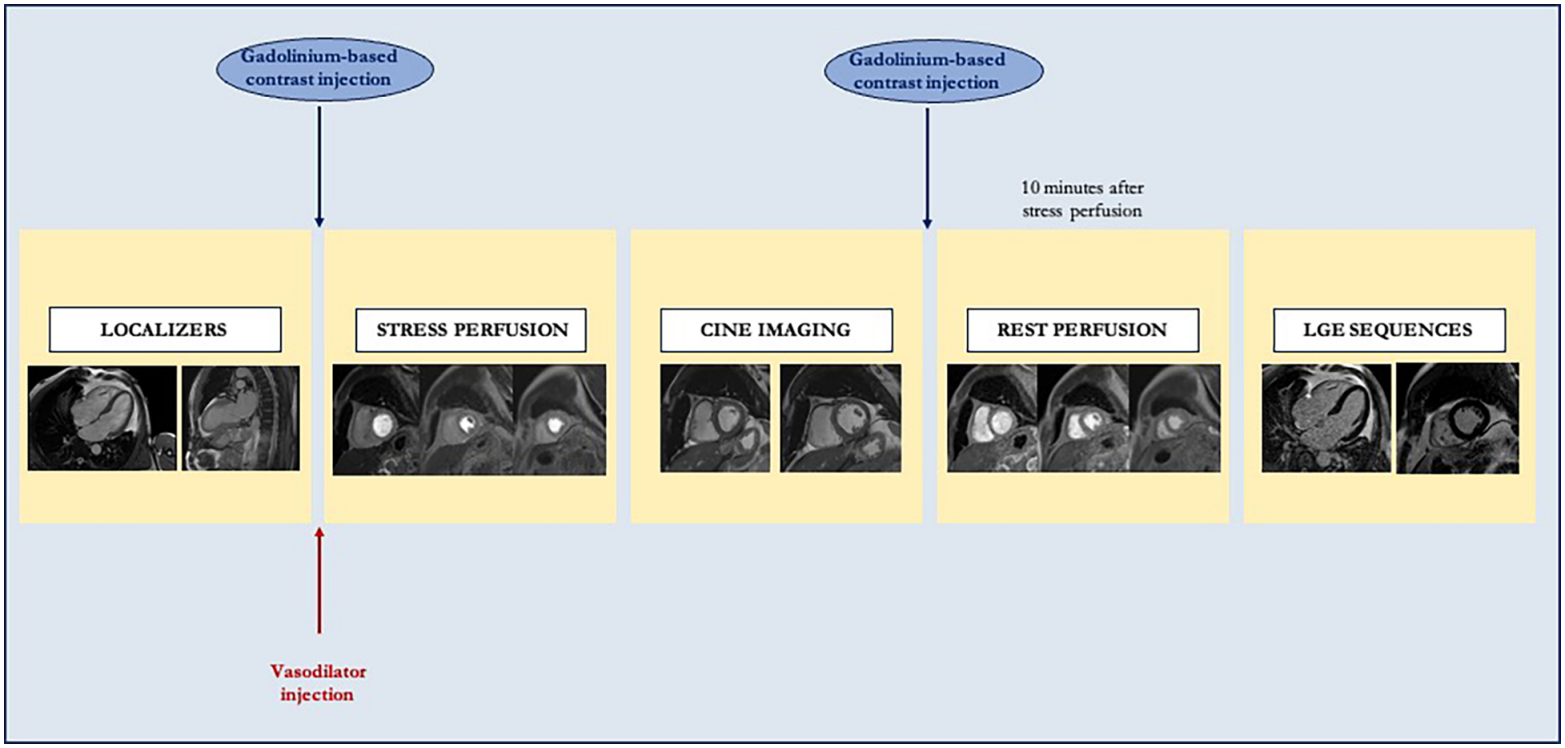
Standard stress cardiac magnetic resonance study protocol. After localizer acquisition, a vasodilator is administered to achieve adequate myocardial stress. Consequently, stress perfusion images are acquired in a three-slice short axis, and gadolinium-based contrast is injected. After stress, cine imaging is acquired. Finally, rest first-pass perfusion imaging and late gadolinium enhancement sequences are acquired to assess viability.

Stress CMR is based on the identification of signal changes of contrast agents that pass through the cardiac chambers and myocardium during dynamic contrast-enhanced perfusion imaging (Figure 2). To evaluate the efficacy of myocardial perfusion in response to increased metabolic requests, it is essential to compare the stress and rest images. Myocardial stress imaging is obtained with intravenous vasodilator administration, commonly using adenosine, dipyridamole, regadenoson, or adenosine triphosphate (66). Each vasodilator agent presents peculiar pharmacokinetic and hemodynamic properties, so the choice depends on local preferences. Adenosine, commonly used for echocardiographic CFVR evaluation, requires a continuous infusion based on the patient's weight (140 μg/kg/min) in an intravenous catheter, different from that used for contrast administration. Adenosine increases coronary blood flow approximately 3–5-fold and is contraindicated in patients with asthma and advanced atrioventricular block (68). Dipyridamole requires a 4 min infusion at a dose of 0.56 mg/kg. Conversely to adenosine, dipyridamole is characterized by a longer half-life with a consequent prolonged duration of side effects and less reproducible vasodilation (69). Regadenoson presents the advantage of using a non-weight-based fixed dose (400 μg) with a half-life of 20 min (66). Due to its longer half-life, regadenoson could require aminophylline administration to easily terminate the side effects of vasodilation, as well as for dipyridamole. Different from adenosine, regadenoson is contraindicated only for patients with advanced atrioventricular blocks, which is safe in asthma patients. Due to its properties, regadenoson is the most used vasodilator in clinical practice. Adenosine triphosphate shares with adenosine the same hemodynamic effects, requiring a slightly longer infusion, but it is used mainly in the Asian–Pacific regions (68).

During vasodilator administration, an evaluation of correct myocardium activation, in terms of an effective increase of cardiac metabolic requests, is mandatory. In fact, an accurate stress CMR is based not only on a correct imaging acquisition but also on an effective increase of myocardium metabolic requests, responsible for a greater coronary blood flow. An increase in heart rate of >10 bpm or a reduction of systolic blood pressure of >10 mmHg with classical vasodilation-induced symptoms (palpitation, dyspnea) is considered markers of an efficient hyperemic response after 2–3 min of vasodilator agent infusion (66). During adenosine infusion, the evidence of a splenic switch-off represents a sign of an appropriate response to the vasodilator. In fact, adenosine plays an action on the A1/A2B receptors in the splenic blood vessels, producing vasoconstriction and a consequent reduction of spleen intensity (70). In the presence of an inadequate hyperemic response, an increase in adenosine dose (up to 210 μg/kg/min) could be considered to reach evaluable myocardial stress (71).

After vasodilator administration, gadolinium-based contrast is injected (0.2 mmol/kg of body weight), followed by saline flush (≥30 ml) into a peripheral vein. Therefore, the distribution of gadolinium-based contrast into cardiac chambers and, consequently, to myocardium allows the evaluation of early myocardial perfusion with electrocardiogram-gated fast T1-sensitive sequences, performed in stress and, subsequently, in the rest (72). In these sequences, an impaired perfusion is responsible for a slow contrast agent diffusion and a reduced T1 signal, in comparison to normal segments.

After 10 min from the stress myocardial perfusion imaging, the rest perfusion imaging could be acquired. In particular, the rest perfusion sequences should be performed with the same image position and the same dose of gadolinium-based contrast, without vasodilator agent administration. Cine sequence images are usually obtained between stress and rest imaging perfusion acquisition.

**Figure 3:**
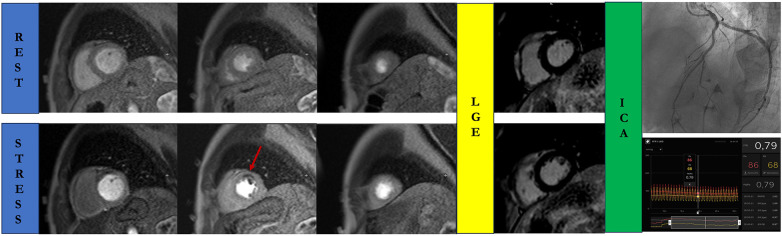
Stress cardiac magnetic resonance in a 68-year-old man with atypical chest pain and negative T waves in the anterior leads. A true perfusion defect is visible as subendocardial hypointense stria (red arrow) in the anterior wall in the stress perfusion imaging, in the absence of myocardial fibrosis in the delayed post-contrast sequences. Rest perfusion images do not reveal any perfusion defects. Invasive coronary angiography confirms the presence of a functionally significant left anterior descending artery disease. ICA, invasive coronary angiography; LGE, late gadolinium enhancement.

Stress perfusion CMR is usually interpreted qualitatively in routine clinical practice, comparing stress and rest images to identify a true perfusion defect (67). A true perfusion defect is characterized by a persistent hypointensity of >5 RR intervals beyond peak myocardial enhancement across more than two pixels. Depending on the extension of the perfusion defect, this persistent hypointensity could be subendocardial or transmural with the involvement of the entire wall thickness, following the coronary distribution (67). A perfusion defect presents only in the stress perfusion imaging could be likely due to a true hypoperfusion, secondary to a coronary stenosis (Figure 3). Conversely, a transient and less than one-pixel-wide hypointensity, which appears when contrast arrives in the left ventricular cavity but before myocardial enhancement, could be due to a dark rim artifact.

LGE sequences are acquired 10 min after the rest perfusion acquisition and allow us to identify the presence of myocardium fibrosis and assess viability. An ischemic LGE pattern is characterized by a subendocardial or transmural hyperintense stria, depending on the extension of the ischemic process. The wall thickness extension of LGE, as well as the number of segments involved in the ischemic process, has an important prognostic value. In particular, Kim et al. (73) demonstrated that the change of recovery in cardiac function was 60%, 40%, 10%, and 1% for wall thickness infarcts involving 1%–25%, 26%–50%, 51%–75%, and >75% thickness infarct, respectively. Perfusion defect, in the absence of LGE, could be due to a dysfunctional myocardium secondary to stunning or hibernation (74).

## Semiquantitative and quantitative myocardial perfusion analysis with stress CMR

**Figure 4:**
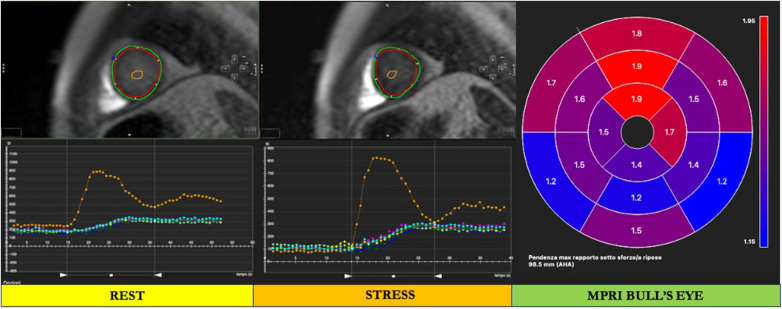
Cardiac magnetic resonance semiquantitative myocardial perfusion assessment. After loading stress and rest perfusion imaging in the dedicated module, the endocardium and epicardium are manually traced in both rest and stress perfusion images (red and green contours, respectively). A segment of the left ventricular (LV) blood cavity, with the exclusion of papillary muscles, is traced in each image (orange contour in the LV cavity). To provide LV myocardial segmentation, the superior and inferior insertion points of the right and left walls are labeled (blue and pink points, respectively). Finally, the signal intensity of the myocardium and LV blood pool are automatically generated by the software, and a bull's eye is provided. In the bull's eye, the myocardial perfusion reserve index (MPRI) is automatically derived by the software.

Patients with CMD usually present coronary arteries free of significant lesions. The absence of an inducible perfusion defect has excellent accuracy in identifying low-risk patients with known or suspected CAD (75). However, the absence of a perfusion defect is not synonymous with normal coronary flow because it could hide a CMD. Myocardial perfusion in stress CMR could be evaluated with visual or quantitative assessment. Visual assessment of myocardial perfusion in CMD patients demonstrated a sensitivity of 41% (95% CI: 27%–57%) (76). Thus, a more accurate and reproducible evaluation of myocardial perfusion could be obtained with quantitative assessment. Semi- and fully quantitative methods revealed high sensitivity and specificity, allowing a more accurate evaluation of CMD (77–79). These quantitative methods are based on the signal intensity during the first pass of gadolinium-based contrast to the myocardium. The evaluation of the signal intensity profile is related to semiquantitative methods and allows us to evaluate the myocardial perfusion reserve index (MPRI), using a dedicated post-processing software (67). After loading stress and rest perfusion imaging in the dedicated module, the endocardium and epicardium are manually traced in the basal, mid, and apical slices of both stress and rest perfusion images. A segment of the LV blood cavity, with the exclusion of papillary muscles, is traced in each image. To provide LV myocardial segmentation, the superior and inferior insertion points of the right and left walls are labeled. Finally, the signal intensity of the myocardium and LV blood pool are automatically generated by the software. MPRI is a robust semiquantitative imaging marker that represents the vasodilatory capacity of the coronary microcirculation in response to a vasodilator stress (Figure 4) (80, 81). MPRI is calculated as the ratio between stress and rest upslope normalized to the upslope of the LV blood pool (82, 83). Similar to echocardiography and PET, a reduced MPRI is a sign of reduced coronary vasodilation in response to hemodynamic stress and could be useful for the CMD diagnosis (84, 85). A global MPRI of ≥2.0 is considered normal (86, 87).

Several evidence showed that CMD patients presented an unfavorable outcome with a greater risk of cardiovascular death, non-fatal myocardial infarction, non-fatal stroke, and hospitalization due to heart failure or unstable angina (88–90). Reduced myocardial perfusion assessed by stress CMR has been demonstrated to be predictive of MACEs in a women’s cohort with myocardial ischemia without significant coronary artery lesions, suggesting its prognostic role (91). Furthermore, Zhou et al. (86) demonstrated that an MPRI of ≤1.47 can predict MACEs in CMD patients (HR = 3.14; 95% CI: 1.58–6.25; *p* = 0.001). Therefore, MPRI emerged as a useful diagnostic and prognostic marker of impaired myocardial perfusion, in the absence of significant coronary stenosis in CMD patients, helping clinicians in patient risk stratification.

Finally, stress CMR could also estimate the myocardial blood flow (MBF) in units of milliliters of blood per minute per gram (ml/min/g), allowing a fully quantitative analysis of myocardial perfusion. To correctly estimate the MBF, an accurate measurement of the arterial input function is crucial, using a dual-bolus method or a dual-sequence technique (81, 92).

## MPRI in ARDs patients

CVD strongly impacts the long-term prognosis of ARDs patients. Therefore, the identification of CMD, as a significant determinant of increased CV risk, becomes crucial to address the best therapeutic management and clinical follow-up for patients with ARDs. As previously described, several non-invasive and invasive methods could be used to identify CMD. Recently, stress CMR revealed its potential to evaluate myocardial ischemia, perfusion, and tissue characterization, avoiding the well-known echocardiography limitations in image acquisition. MPRI has been widely evaluated in ARDs patients with promising results. Chen et al. (17) have demonstrated that among women with CMD (19/207 patients), patients with ARDs presented reduced MPRI (*p* = 0.008), not captured by the echocardiographic CFVR (*p* = 0.07). Confirming this result, MPRI has been evaluated in many ARDs (Table 1).

**Table 1:** Myocardial perfusion reserve index performed by cardiac magnetic resonance in ARDs patients.

Author, study	Disease	Vasodilator stressor used	Study—aims	Patients included	Results
Chen MT, et al. Frontiers in Cardiov Medicine (17)	ARDs	Adenosine/regadenoson	Determine MPRI in women with CMD and ARDs	207 women with CMD: 19 women had ARDs	Women affected by ARDs had lower functional capacity and lower MPRI
Ishimori ML, et al. JACC Cardiovascular Imaging (93)	Systemic lupus erythematosus (SLE)	Adenosine/regadenoson (in two asthma patients)	Evaluate the presence of myocardial perfusion defect and MPRI in female SLE patients	– 20 SLE female with typical and atypical chest pain – 10 asymptomatic reference control womenMPRI was evaluated globally, in the subendocardium and subepicardium	– SLE patients had lower subepicardial MPRI compared to controls – SLE was the only predictor of subepicardial MPRI
Sandhu VK, et al. Arthritis Care Res (94)	Systemic lupus erythematosus (SLE)	Adenosine	Evaluate the serial changes in chest pain, CMD, and obstructive CAD in patients with SLE in a 5-year follow-up study	20 SLE females with chest pain and no obstructive CAD by CCTA who underwent stress CMR → were re-evaluated at 5 years	– 11/17 had persistent chest pain → 5/14 had CMD on follow-up
Mavrogeni S, et al. International Journal of Cardiology (95)	Systemic sclerosis (SS)	Adenosine	Evaluate myocardial perfusion–fibrosis in SS using CMR (MPRI and LGE)	– 7 asymptomatic SS patients (5 with diffuse and 2 with limited SS) – 7 controls – 5 patients with CAD	– Non-segmental, subendocardial perfusion defects were identified in all SS patients – Segmental, subendocardial perfusion defects were identified in 3/5 CAD – The lowest MPRI in patients with diffuse SS – LGE in SS was diffuse
Mavrogeni SI, et al. Inflammation and allergy, (96)	Systemic sclerosis (SS)	Adenosine	Evaluate inflammation, myocardial perfusion, and fibrosis in diffuse systemic scleroderma. Two-year follow-up	– 46 asymptomatic patients with diffuse SS – 20 controls – 20 patients with CAD	– 44/46 had lower MPRI compared to controls – LGE was diffuse and greater than controls, but not in comparison with CAD patients – In follow-up (available in 11/44 patients) SS patients presented further MPRI deterioration and diffuse subendocardial fibrosis
Mavrogeni S, et al. IJC (97)	Peripheral Raynaud's phenomenon	Adenosine	Evaluate myocardial perfusion in patients with peripheral Raynaud's phenomenon	– 20 RP due to connective tissue diseases – 20 patients with primary RP – 20 controls	– MPRI was lower in RP patients than in controls – Patients with secondary RP had a more severe reduction of MPRI
Mavrogeni SI et al. J of Clinical Medicine, (98)	Antiphospholipid syndrome (APS)	Adenosine	Determine the prevalence of silent myocardial ischemia and fibrosis in patients with APS and SLE/APS without known CAD Identify potential association between CMR findings and APS-related and classic CVD risk factors and coronary angiography findings 12-month follow-up	– 44 patients with APS without prior cardiac disease → 22 with primary APS and 22 with SLE/APS – 44 age-/gender-matched controls	– Median MPRI was lower in APS than controls, independently of LGE – LGE was present in 16/44 APS patients → 16 patients underwent coronary angiography → only 2/16 had CAD – At follow-up, 3/44 patients had CAD (presented the lowest MPRI values)

APS, antiphospholipid syndrome; ARDs, autoimmune rheumatic diseases; CAD, coronary artery disease; CCTA: coronary computed tomography angiography; CMD, coronary microvascular dysfunction; CMR, cardiac magnetic resonance; CVD, cardiovascular; LGE, late gadolinium enhancement, MPRI, myocardial perfusion reserve index; RP, Raynaud's phenomenon, SLE, systemic lupus erythematous; SS, systemic sclerosis.

SLE is a systemic autoimmune disorder, more prevalent in females, characterized by chronic and systemic inflammation. Morbidity and mortality in SLE are mainly due to cardiac manifestations, especially CAD and myocarditis (10). SLE patients demonstrated to have a 7.5-fold increased risk of developing CAD in comparison to the general population (10). Although an accelerated atherosclerotic process could partially explain this increased CV risk, myocardial ischemia potentially due to CMD has been hypothesized. A significant CFVR reduction has been reported in young women affected by SLE, confirming the presence of coronary microvascular impairment (99, 100). Accordingly, an impaired microvascular perfusion visually assessed with stress CMR has been identified in a small cohort of 20 SLE female patients with chest pain and non-obstructive CAD on CCTA (93). In this study, SLE emerged as the only determinant of a reduced subepicardial MPRI, which has been identified in patients when compared to the control group (2.0 ± 0.4 vs. 2.4 ± 0.4; *p* = 0.031). Sandhu et al. (94) evaluated the prognostic role of MPRI in a small group of SLE female patients with chest pain who underwent CCTA and stress CMR at baseline and after 5 years. This study demonstrated that after 5-year of follow-up, the majority of SLE patients had persistent angina (11/17 patients) and nearly half had similar or worsened MPRI compared to the baseline, confirming that CMD represents a major cause of persistent chest pain in the absence of obstructive coronary lesions in this subset of patients.

Systemic sclerosis is a connective tissue disease characterized by vascular dysfunction, autoimmunity, and increased fibroblast activity, responsible for systemic diffuse fibrosis (95). Cardiac involvement is mainly due to the fibrinoid necrosis of intramural coronaries, detected by pathology, that determines diffuse myocardial fibrosis, hypoperfusion, and, consequently, CMD (96). The absence of significant epicardial coronary stenosis confirms that the high microcirculation resistance, secondary to vascular fibrosis, compromises myocardial perfusion (101). Accordingly, CMR widely reported diffuse and non-segmental myocardial fibrosis, not related to coronary artery distribution, as a manifestation of diffuse hypoperfusion (95, 102, 103). As an expression of impaired coronary microvasculature, a reduced CFVR has been found in about 50%–60% of clinically scleroderma patients (104, 105). Mavrogeni et al. (95) showed that SS patients had lower MPRI in comparison with CAD patients (1.2 ± 0.5 vs. 1.8 ± 0.2) and, consequently, than controls (1.2 ± 0.5 vs. 2.46 ± 0.3, *p* < 0.001). After 2 years of follow-up, a further reduction of MPRI from the baseline has been reported (0.5 ± 0.1 vs. 0.9 ± 0.2), in the absence of significant morpho-functional modifications, confirming the pivotal role of CMD in the cardiac involvement in SS patients (103). A potential role of nifedipine in the improvement of myocardial perfusion has been advocated, suggesting a possible reversibility of the perfusion defect (106). Likewise, Allanore et al. (107) described an interesting role of bosentan in myocardial perfusion and function in SS patients. Similar to other ARDs, cardiac involvement, in terms of CMD, has been associated with a poor prognosis (101, 108).

Peripheral Raynaud's phenomenon, first reported by Raynaud in 1892 as episodic digital ischemia in response to cold exposure or emotion in the absence of any arterial occlusion, could be associated with cardiac manifestations (97, 109). Similarly to CMD, Raynaud's phenomenon involves mainly young women (110), and it could be primary or secondary to other ARDs or connective tissue disorders (111). As a vasospastic disorder responsible for color and trophic skin changes, coronary microvasculature has been demonstrated to be strongly involved in the natural history of the disease. In fact, recently, a lower MPRI has been reported in patients with Raynaud's phenomenon, both for primary and secondary forms, in comparison to the control group (1.7 ± 0.6 vs. 3.5 ± 0.4, *p* < 0.001, and 0.7 ± 0.2 vs. 3.5 ± 0.4, *p* < 0.001, respectively). Patients with secondary Raynaud's phenomenon seem to have a more severe coronary microvasculature impairment in comparison with those with primary one (0.7 ± 0.2 vs. 1.7 ± 0.6, *p* < 0.001) (97).

Finally, patients affected by antiphospholipid syndrome (APS) have been demonstrated to have CMD. APS is a rare systemic autoimmune disease, characterized by vascular thrombosis, pregnancy morbidity, and persistent positive APL antibodies, often associated with SLE (as secondary APS) (1, 112). Thus, according to other ARDs, APS patients presented lower MPRI values when compared to controls (1.5 vs. 2.7, *p* < 0.001), regardless of the presence of myocardial fibrosis. Myocardial fibrosis was detected in one-third of APS patients (16/44 patients); twelve patients underwent coronary angiography, revealing CAD in only two patients. Interestingly, in the 12-month follow-up, three patients with the lowest MPRI values experienced ischemic events (one patient with myocardial infarction and two patients with unstable angina) (98). Thus, MPRI was able to identify CMD and, interestingly, the lowest value resulted associated with MACEs, revealing a prognostic role in APS patients.

Although MPRI has been studied in a small cohort of ARDs patients, reflecting the real-world prevalence of these diseases, the precocious identification of abnormal myocardial perfusion could have important clinical repercussions. Firstly, stress CMR emerges as a valuable and reproducible non-invasive tool, able to identify CMD in ARDs patients. Secondly, the recognition of cardiac involvement in ARDs patients without angina-related symptoms allows us to start therapy early, aiming to reduce the CMD’s negative prognostic role on MACEs. Although a specific therapy for CMD in ARDs patients has not been reported, the beneficial role of antianginal and anti-atherosclerotic therapy in CMD patients has been extensively described (113). In fact, beta-blockers, nitrates, and calcium-channel blockers are widely demonstrated to improve angina symptoms and exercise capacity in CMD patients (114). Beta-blockers are considered the first-line therapy for CMD patients for their role in reducing adrenergic activity and myocardial oxygen demand through the NO-mediated vasodilatory effect (16). Nitrates could be considered in patients with acute anginal episodes and an abnormal vasodilatory reserve (115). Finally, calcium-channel blockers are the first-line therapy for patients with vasospasm-mediated CMD (115, 116). Moreover, considering the possible overlap of CMD with atherosclerosis, the use of angiotensin-converting enzyme inhibitors or receptor blockers, statins, and aspirin revealed a beneficial effect, in terms of endothelial function improvement, plaque, and oxidative stress reduction and anti-inflammation role in CMD patients (113).

Finally, non-pharmacologic treatment including exercise, weight loss, and smoking cessation has been demonstrated to improve CFR and angina-related symptoms in CMD patients (117, 118).

Therefore, based on these promising results and the potential clinical repercussions of an early CMD diagnosis, the role of MPRI should be further investigated to establish its validity also in other clinical settings.

## Conclusions

Patients with ARDs present an increased CV risk, not fully explained by atherosclerotic progression. CMD emerges as a crucial determinant of CV risk in these patients, as the result of inflammation, endothelial dysfunction, oxidative stress, and autoimmunity. Although several non-invasive and invasive imaging tests can identify CMD, stress CMR has been demonstrated to accurately assess myocardial ischemia, viability, and CMD in ARDs patients. Early CMD diagnosis in asymptomatic patients may allow us to start a precocious therapy to significantly impact the natural history of the disease.
